# Dopamine-induced arrestin recruitment and desensitization of the dopamine D4 receptor is regulated by G protein-coupled receptor kinase-2

**DOI:** 10.3389/fphar.2023.1087171

**Published:** 2023-01-27

**Authors:** Viktor Burström, Richard Ågren, Nibal Betari, Marta Valle-León, Emilio Garro-Martínez, Francisco Ciruela, Kristoffer Sahlholm

**Affiliations:** ^1^ Department of Integrative Medical Biology, Wallenberg Centre for Molecular Medicine, Umeå University, Umeå, Sweden; ^2^ Department of Neuroscience, Karolinska Institutet, Solna, Sweden; ^3^ Pharmacology Unit, Department of Pathology and Experimental Therapeutics, Faculty of Medicine and Health Sciences, Institute of Neurosciences, University of Barcelona, Barcelona, Spain; ^4^ Neuropharmacology and Pain Group, Neuroscience Program, Institut d'Investigació Biomèdica de Bellvitge, IDIBELL, Barcelona, Spain

**Keywords:** luminescence measurements, luciferase, electrophysiology, HEK 293 cells, *Xenopus laevis*, G protein-coupled inwardly rectifying potassium channels

## Abstract

The dopamine D_4_ receptor (D_4_R) is expressed in the retina, prefrontal cortex, and autonomic nervous system and has been implicated in attention deficit hyperactivity disorder (ADHD), substance use disorders, and erectile dysfunction. D_4_R has also been investigated as a target for antipsychotics due to its high affinity for clozapine. As opposed to the closely related dopamine D_2_ receptor (D_2_R), dopamine-induced arrestin recruitment and desensitization at the D_4_R have not been studied in detail. Indeed, some earlier investigations could not detect arrestin recruitment and desensitization of this receptor upon its activation by agonist. Here, we used a novel nanoluciferase complementation assay to study dopamine-induced recruitment of β-arrestin2 (βarr2; also known as arrestin3) and G protein-coupled receptor kinase-2 (GRK2) to the D_4_R in HEK293T cells. We also studied desensitization of D_4_R-evoked G protein-coupled inward rectifier potassium (GIRK; also known as Kir3) current responses in *Xenopus* oocytes. Furthermore, the effect of coexpression of GRK2 on βarr2 recruitment and GIRK response desensitization was examined. The results suggest that coexpression of GRK2 enhanced the potency of dopamine to induce βarr2 recruitment to the D_4_R and accelerated the rate of desensitization of D_4_R-evoked GIRK responses. The present study reveals new details about the regulation of arrestin recruitment to the D_4_R and thus increases our understanding of the signaling and desensitization of this receptor.

## 1 Introduction

Dopamine is involved in the regulation of selective attention, memory, and learning, reward-driven behavior, and voluntary movement (Missale et al., 1998; Beaulieu and Gainetdinov, 2011). Dopamine receptors are G protein-coupled receptors (GPCRs) and come in five different subtypes, with D_1_-like receptors (D_1_R and D_5_R) coupling to stimulatory (at the level of adenylate cyclase modulation) Gα_s/olf_ proteins and D_2_-like receptors (D_2_R, D_3_R, and D_4_R) coupling to inhibitory Gα_i/o_ proteins. The D_4_R is encoded by the *DRD4* gene, which is expressed most abundantly in the retina, with moderate expression in brain tissue (Matsumoto et al., 1995; Meador-Woodruff et al., 1996). Within the brain, notable D_4_R expression sites include GABAergic interneurons in the prefrontal cortex, as well as cortical glutamatergic pyramidal neurons and their corticostriatal terminals (Lauzon and Laviolette, 2010; González et al., 2012; Bonaventura et al., 2017). Using D_4_R knockout mice, the relative contribution of the D_4_R to total D_2_-like receptor expression has been estimated to 17% in the ventral striatum, 21% in the caudate-putamen and olfactory tubercle, and 40% in the hippocampus (Defagot et al., 2000). The D_4_R shares 41% and 39% sequence identity with the D_2_R and the D_3_R, respectively (Van Tol et al., 1991) and contains a region of variable number tandem repeats (VNTRs), giving rise to imperfect 16-residue repeats in its third intracellular loop (Van Tol et al., 1992; Lichter et al., 1993). While a four-repeat variant is the most common in the global population, two- and seven-repeat variants are also common, with considerable geographical differences in the relative abundance of these variants (Van Tol et al., 1992; Lichter et al., 1993). The seven-repeat variant has been associated with an increased risk of attention deficit hyperactivity disorder (ADHD) in several studies (Li et al., 2006; Bonvicini et al., 2020), suggesting a role for this receptor in the regulation of selective attention. The D_4_R has also been proposed as a therapeutic target for substance use disorders and erectile dysfunction (Di Ciano et al., 2014; Wang et al., 2017). Moreover, the D_4_R is known to regulate autonomous nervous system activity (García-Pedraza et al., 2020) and has a similar affinity for both noradrenaline and dopamine (Sánchez-Soto et al., 2016).

Arrestins are proteins that interact with the intracellular loops and C-terminal regions of GPCRs. Arrestins typically compete with G proteins for receptor binding and sometimes initiate receptor internalization [16], leading to desensitization of G protein-dependent signaling. However, arrestins can also scaffold a host of other signaling proteins, thus setting off a distinct set of signaling events. Many GPCRs, including D_2_-like receptors, are known to signal *via* both the classical G protein-dependent pathways and the more recently described arrestin pathways (Beaulieu and Gainetdinov, 2011; Wingler and Lefkowitz, 2020). As their name implies, G protein-coupled receptor kinases (GRKs) are able to phosphorylate GPCRs, often agonist-dependently (Kim et al., 2001). This phosphorylation has been found to increase the abilities of several GPCRs, including the D_2_R (Pack et al., 2018; Mann et al., 2021), to recruit arrestins. While there are several GRK isoforms, GRK2 is considered an important regulator of D_2_R signaling in the cerebral cortex (Urs et al., 2016).

Similarly to D_2_R and D_3_R, the coupling of D_4_R to inhibitory G proteins and consequent inhibition of adenylate cyclase and opening of G protein-coupled inward rectifier potassium (GIRK; also known as Kir3) channels have been repeatedly demonstrated (Chio et al., 1994; Asghari et al., 1995; Werner et al., 1996; Liu et al., 1999; Kazmi et al., 2000; Sahlholm et al., 2008). In contrast, while both D_2_R and D_3_R are known to recruit arrestins upon activation, there have been conflicting reports on the ability of D_4_R to interact with this class of signaling proteins. Indeed, some previous investigations could not detect significant agonist-induced arrestin recruitment to D_4_R, which was suggested to be “non-desensitizing” (Spooren et al., 2010; Forster et al., 2020; Zheng et al., 2020)**.** Furthermore, little is known about the putative regulation of D_4_R signaling by GRKs. Here, we used a novel nanoluciferase complementation assay in HEK293T cells, as well as GIRK response desensitization in *Xenopus* oocytes, to investigate βarr2 recruitment to D_4_R and its modulation by GRK2 coexpression.

## 2 Materials and methods

### 2.1 Molecular biology

D_4_R-NP (‘native peptide’) and D_2_R-NP (a gift from Drs. Julien Hanson and Céline Laschet, University of Liège, Belgium) were in pcDNA3.1+ (Thermo Fisher Scientific, Waltham, MA). D_4_R-NP was designed to be analogous to D_2_R-NP, which was previously described by Laschet et al. (Laschet et al., 2019). Thus, in addition to the C-terminal linker (GNSGSSGGGGSGGGGSSG) and NP tag (GVTGWRLCERILA), D_4_R-NP contained an N-terminal cleavable influenza hemagglutinin signal peptide (KTIIALSYIFCLVFA) to promote surface expression, followed by a FLAG tag (DYKDDDDK). For oocyte experiments, D_4_R, RGS4, GIRK1 (Kir3.1), GIRK4 (Kir3.4), and GRK2 were in pXOOM (provided by Dr. Søren-Peter Olesen, University of Copenhagen, Denmark). Both D_4_R constructs were based on the four-repeat variant of human D_4_R (D_4.4_R) and were, along with GRK2-LgBiT (LgBiT fused directly C-terminally to human GRK2) and untagged human βarr2 and GRK2, synthesized by Genscript, Inc. (Piscataway, NJ) and cloned into pXOOM. LgBiT-βarr2 (rat βarr2 N-terminally fused to LgBiT.

VFTLEDFVGDWEQTAAYNLDQVLEQGGVSSLLQNLAVSVTPIQRIVRSGENALKIDIHVIIPYEGLSADQMAQIEEVFKVVYPVDDHHFKVILPYGTLVIDGVTPNMLNYFGRPYEGIAVFDGKKITVTGTLWNGNKIIDERLITPDGSMLFRVTINS,

in pNBe3, Promega, Madison, WI) was also a gift from Drs. Julien Hanson and Céline Laschet. For oocyte experiments, the plasmids were linearized using the appropriate restriction enzymes (D_4_R, GIRK1, GIRK4, and RGS4; XhoI and βarr2 and GRK2; XbaI), followed by *in vitro* transcription using the T7 mMessage mMachine kit (Ambion, Austin, TX). The concentration and purity of cRNA were determined by spectrophotometry.

### 2.2 Luciferase complementation assay

We adapted a nanoluciferase assay described by Laschet et al. (2019) to measure the interaction between D_4_R and downstream signaling proteins. HEK293T cells (a gift from Drs. Per Svenningsson and Xavier Morató Arús, Karolinska Institutet, Stockholm, Sweden) were incubated at 37 °C with 5% CO2 in 10 cm culture plates (VWR part of Avantor, Radnor, PA) containing Dulbecco’s modified eagle medium (DMEM; Thermo Fisher Scientific) supplemented with 0.01% penicillin/streptomycin (Thermo Fisher Scientific) and 10% FBS (Thermo Fisher Scientific). Cells were transfected with 1 µg/dish D4R-NP or D2R-NP and 1 µg/dish LgBiT-βarr2/GRK2-LgBiT along with 10 µg untagged GRK2, when indicated, using linear polyethylenimine (PEI; Polysciences Inc., Valley Road Warrington, PA (Longo et al., 2013). The empty plasmid vector (pcDNA3.1+) was added to the transfection mixture to bring the total amount of transfected plasmid to 20 µg/dish. Subsequently, 24 h after transfection, cells were trypsinated and centrifugated for pellet recovery before resuspension in Hank’s balanced salt solution (HBSS; Corning, Tewksbury, MA). Cells were counted using a TC20 automated cell counter (Bio-Rad, Hercules, CA) and diluted in HBSS at 500,000 cells/ml. 100 µl cell solution was pipeted into each well of a flat-bottom white 96-well plate (Thermo Fisher Scientific). The nanoluciferase substrate furimazine was added to each well in the form of Nano-Glo live cell reagent (Promega) according to the manufacturer’s instructions. For concentration-response experiments, serial dilutions of dopamine (Sigma-Aldrich, St. Louis, MO) were added to the 96-well plate column-wise, with the first column receiving only vehicle (HBSS). The plate was incubated at room temperature for 5 min (or 3 min in experiments with GRK2-LgBiT) prior to luminescence measurement in a TriStar2 LB 942 multimode reader (Berthold Technologies, Bad Wildbad, Germany) with an integration time of 10 ms.

For time-resolved, repeated measurements, luminescence was recorded from each well each min for 1 h with an integration time of 10 ms. Following a baseline read of 8 min, 10 µL of dopamine dissolved in HBSS was injected into each well to result in a final concentration of 32 nM. After another 10 min, 10 µL of clozapine or raclopride (Tocris Bioscience, Bristol, UK), dissolved in DMSO and diluted in HBSS, were injected to yield a final concentration of 10 µM (clozapine) or 1 µM (raclopride). HBSS injection was used as control.

### 2.3 Oocyte preparation

The oocytes of the African clawed toad, *Xenopus laevis*, were surgically isolated as previously described (Sahlholm et al., 2011). The surgical procedures were approved by the Swedish National Board for Laboratory Animals and the Stockholm Ethical Committee (approval number 686–2021). After 1 day of incubation at 12°C, the oocytes were injected with 0.3 ng of D_4_R cRNA, 1.4 ng of RGS4 cRNA, 40 pg of each of GIRK1 and GIRK4 cRNA in a volume of 50 nL per oocyte using a Nanoject (Drummond Scientific, Broomall, PA). When applicable, 0.6–19 ng/oocyte of βarr2 and 0.3 ng/oocyte of GRK2 cRNA were also injected.

### 2.4 Electrophysiology methods

Following RNA injection and 6 days of incubation at 12 °C, electrophysiological experiments were performed on the oocytes using the parallel eight-channel, two-electrode voltage-clamp OpusXpress 6000A (Molecular Devices, San José, CA) (Ågren and Sahlholm, 2021). Continuous perfusion, provided by Minipuls three peristaltic pumps (Gilson, Middleton, WI), was maintained at 3.5 mL/min. Data were acquired at a membrane potential of −80 mV and sampled at 156 Hz using OpusXpress 1.10.42 software (Molecular Devices). To increase the inward-rectifier potassium channel current at negative potentials, a high-potassium extracellular buffer was used (64 mM NaCl, 25 mM KCl, 0.8 mM MgCl_2_, 0.4 mM CaCl_2_, 15 mM HEPES, 1 mM ascorbic acid, adjusted to pH 7.4 with NaOH), yielding a K_+_ reversal potential of ∼−40 mV. Ascorbic acid was included to prevent spontaneous oxidation of DA.

### 2.5 Whole-cell enzyme-linked immunosorbent assay (ELISA)

Cell surface expression of D_4_R and D_2_R constructs was evaluated essentially as described by (Kozielewicz et al., 2020). In brief, following 24 h incubation after transfection (as described above), cells were trypsinated, resuspended in DMEM (supplemented as above), counted and re-plated at 50,000 cells/well in a volume of 100 µL/well in transparent 96-well plates (Thermo Fischer Scientific) previously coated with poly-D-lysine (Sigma-Aldrich) and returned to the incubator for further growth and adhesion. After another 24 h incubation, DMEM was aspirated and wells were washed twice with 100 µL/well of chilled (4°C) phosphate-buffered saline (PBS; VWR part of Avantor) containing 0.5% (w/v) bovine serum albumin (BSA; Sigma-Aldrich). After washing, cells were incubated with 50 µL/well of horseradish peroxidase-linked mouse anti-FLAG M2 antibody (A8592; Sigma-Aldrich) diluted 1/20,000 with chilled PBS containing 1% BSA. Following 1 h incubation at 4°C, well contents were aspirated and washed four times with chilled PBS containing 0.5% BSA. Next, 50 µL/well of the horseradish peroxidase substrate 3,3′,5,5′-Tetramethylbenzidine (T0440; Sigma-Aldrich) was added and plates were incubated at 37°C for 20 min. Finally, 50 µL/well of 2 M HCl were added to each well and absorbance was measured at 450 nm in the Berthold TriStar2 LB 942 plate reader.

### 2.6 Data analysis

Concentration–response curves for nanoluciferase complementation data were calculated using the variable-slope sigmoidal functions in GraphPad Prism 8 (GraphPad Software, San Diego, CA).

The following equation was used for fitting:

$$Y=1+\left(Top-1\right)/\left(1+10^{\left(\log{EC}_{50}-X\right)}\right)$$ (1)

where *Y* is the response, *Top* is the maximal response as a fraction of the mean control (HBSS) luminescence and *X* is the logarithm of dopamine concentration. Data points are presented as mean ± SEM throughout. pEC50 values from individual experiments were compared using Student’s paired *t*-test, testing differences in pEC50s obtained from experiments performed in the presence or absence of exogenous GRK2. Experiments with and without GRK2 were carried out on the same day with cells of the same passage number.

Electrophysiology recordings were initially screened for inclusion in Clampfit 10.6 (Molecular Devices). Recordings in which holding currents at −40 mV were stable before and after the −80 mV step and in which the current (at −80 mV) after dopamine washout was equal to or smaller than before dopamine application were included in the analysis. Recordings were peak normalized and time-averaged using Matrix Laboratory 2018b (MathWorks, Natick, MA).

### 2.7 Statistical analysis

Statistical analysis was performed in GraphPad Prism 8 using the two-tailed, paried or unpaired Student’s t-test, one-way ANOVA with Dunnett’s test for multiple comparisons, or repeated measures ANOVA with Sidak’s test for multiple comparisons, as appropriate, for normally distributed data. Mann-Whitney test, Wilcoxon matched-pairs signed rank test, or Kruskal–Wallis test with Dunn’s correction for multiple comparisons were used when normal distribution of data could not be assumed. Normality was assessed using the Shapiro-Wilk test. The significance threshold was set to 0.05.

## 3 Results

**FIGURE 1:**
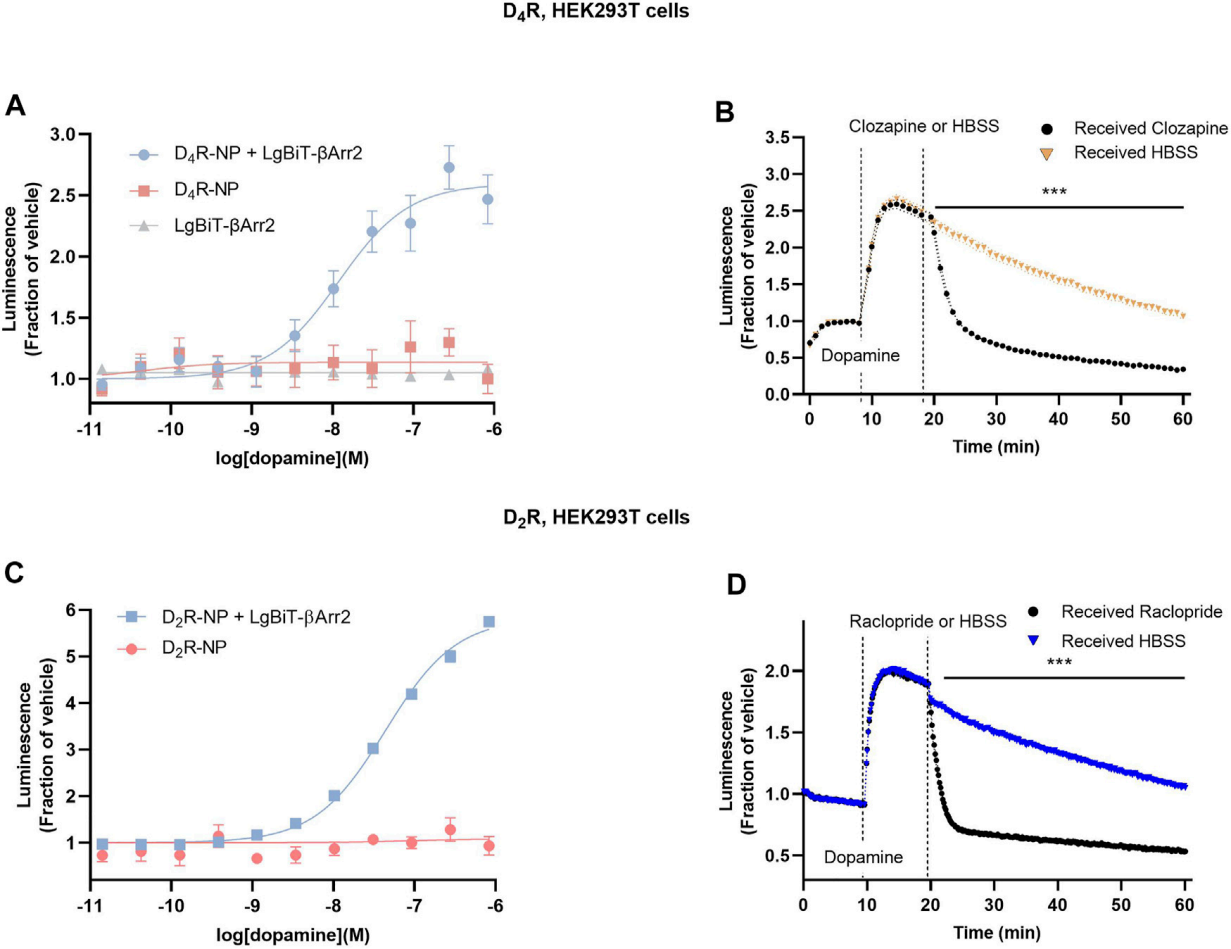
Arrestin recruitment to the D_4_R as measured by a modified NanoBiT complementation bioluminescence assay. **(A)** A dopamine concentration-dependent increase in luminescence was observed in cells co-transfected with D_4_R-NP and LgBiT-βarr2, but not with either construct alone. **(B)** Kinetic experiments revealed a blockade of the dopamine-induced luminescence increase upon application of the D_4_R antagonist clozapine. **(C)** Dopamine concentration-response curve for the increase in luminescence in cells co-transfected with D_2_R-NP and LgBiT-βarr2, or with negative control (D_2_R-NP expressed alone). **(D)** Kinetic experiments revealed a blockade of the dopamine-induced luminescence increase upon application of the D_2_R antagonist raclopride. Asterisks and horizontal bars indicate intervals of statistically significant differences between HBSS control conditions and clozapine or raclopride. ***; *p* < 0.001, repeated measures ANOVA with Sidak’s multiple comparisons test. Luminescence data are expressed as fractions of the signal observed in vehicle-treated wells **(A, C)** or prior to dopamine application **(B, D)**. Each data point in **(A–D)** represents mean ± SEM of eight replicate wells from one representative experiment performed three to five times. SEM bands are shown in **(B, D)**. For some data points, SEM bars or bands are obscured by the symbols representing the means.

First, we designed a nanoluciferase complementation assay to study βarr2 recruitment to D_4_R. To this end, we adapted a modified nanoBiT assay, which was recently demonstrated to work well with D_2_R (Laschet et al., 2019), for use with D_4_R. As the higher-affinity nanoluciferase fragment 'native peptide’ (NP) was reported to result in a higher signal-to-noise ratio compared to the lower-affinity SmBiT (Dixon et al., 2016), we created an analogous D_4_R construct, fusing NP to the C-terminus of D_4_R *via* a flexible linker (*see* Methods). The larger complementary fragment (LgBiT) was fused to the N-terminus of βarr2 (LgBiT-βarr2). In HEK293T cells transfected with D_4_R-NP and LgBiT-βarr2, a concentration-dependent increase in luminescence was observed when cells were stimulated with increasing dopamine in the presence of a constant concentration of the substrate furimazine (Figure 1A). This dopamine-dependent increase was not observed in cells transfected with only LgBiT-βarr2 or only D_4_R-NP (Figure 1A). Kinetic experiments revealed that the increase in luminescence after dopamine application was fully reversible upon the addition of 10 µM of the antipsychotic clozapine, which is a D_4_R antagonist (Van Tol et al., 1991; Lindsley and Hopkins, 2017) (Figure 1B). For comparison, βarr2 recruitment to D_2_R was also studied in a separate series of experiments. As previously described (Laschet et al., 2019), a concentration-dependent increase in luminescence was observed when cells coexpressing D_2_R-NP and LgBiT-βarr2, but not D_2_R-NP alone, were stimulated with increasing dopamine in the presence of furimazine (Figure 1C). In kinetic experiments, the increase in luminescence after dopamine application was fully reversible upon the addition of 1 µM of the D_2_R antagonist raclopride (Figure 1D).

**FIGURE 2:**
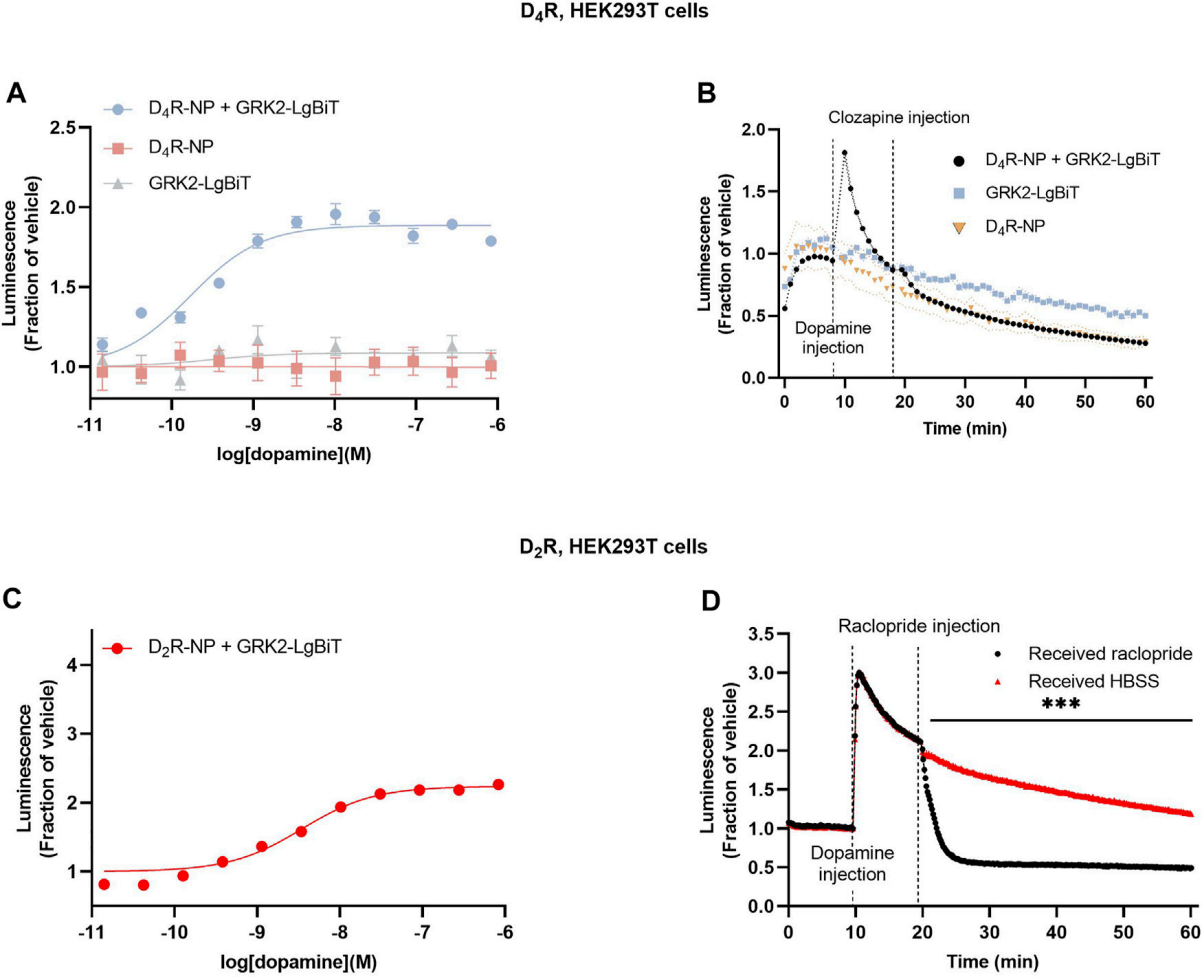
Assessment of GRK2 recruitment to the D_4_R and the D_2_R. **(A)** Concentration-response relationships for dopamine-induce luminescence increase in cells co-expressing D_4_R-NP with GRK2-LgBiT, or either construct expressed alone. Measurements were taken 3 min after the addition of dopamine. **(B)** Kinetic experiments revealed a transient increase in dopamine-induced luminescence in cells co-expressing D_4_R-NP with GRK2-LgBiT. **(C)** Concentration-response relationships for dopamine-induce luminescence increase in cells co-expressing D_2_R-NP with GRK2-LgBiT. Measurements were taken 3 min after the addition of dopamine. **(D)** Kinetic experiments showing the time course of the dopamine-induced luminescence increase in cells co-expressing D_2_R-NP with GRK2-LgBiT and the blockade of the response to dopamine upon application of the D_2_R antagonist raclopride. Asterisks and horizontal bar indicate an interval of statistically significant differences between the raclopride and HBSS control conditions. ***; *p* < 0.001, repeated measures ANOVA with Sidak’s multiple comparisons test. Luminescence data are expressed as fractions of the signal observed in vehicle-treated wells **(A, C)**, or prior to dopamine application **(B, D)**. Data points represent mean ± SEM from eight replicate wells from one representative example out of three independent experiments. SEM bands are shown in **(B, D)**. For some data points, SEM bars or bands are obscured by the symbols representing the means.

Next, we wanted to assess the putative interaction between D_4_R and GRK2 itself. Dopamine application to cells co-expressing D_4_R-NP with LgBiT C-terminally fused to GRK2 (GRK2-LgBiT) revealed a dopamine concentration-dependent increase of luminescence (Figure 2A). Kinetic experiments revealed a transient dopamine-induced bioluminescence signal, which peaked rapidly following dopamine application (Figure 2B). No increase in luminescence was observed upon addition of dopamine to cells expressing GRK2-LgBiT without D_4_R-NP. The luminescence response in cells coexpressing D_4_R-NP and GRK2-LgBiT returned towards baseline (in the continued presence of dopamine) much faster than what was observed in cells co-expressing D_4_R-NP and LgBiT-βarr2 (c.f. Figure 1B; T_1/2_ 2.9 ± 0.3 min vs 18.7 ± 3.5 min, n = 3 in each case; *p* = 0.011, Student’s unpaired *t*-test). For comparison, we also assessed the recruitment of GRK2 to the D_2_R. Similar to the findings with D_4_R-NP, dopamine concentration-dependently increased luminescence in cells transfected with GRK2-LgBiT and D_2_R-NP, although the fold-over-baseline increase in luminescence was greater than with the D_4_R-based construct (Figure 2C). Moreover, in kinetic experiments, the increase in luminescence was more sustained with D_2_R-NP (Figure 2D). Application of the D_2_R antagonist raclopride (1 µM) rapidly reversed the dopamine-induced luminescence increase (Figure 2D).

**FIGURE 3:**
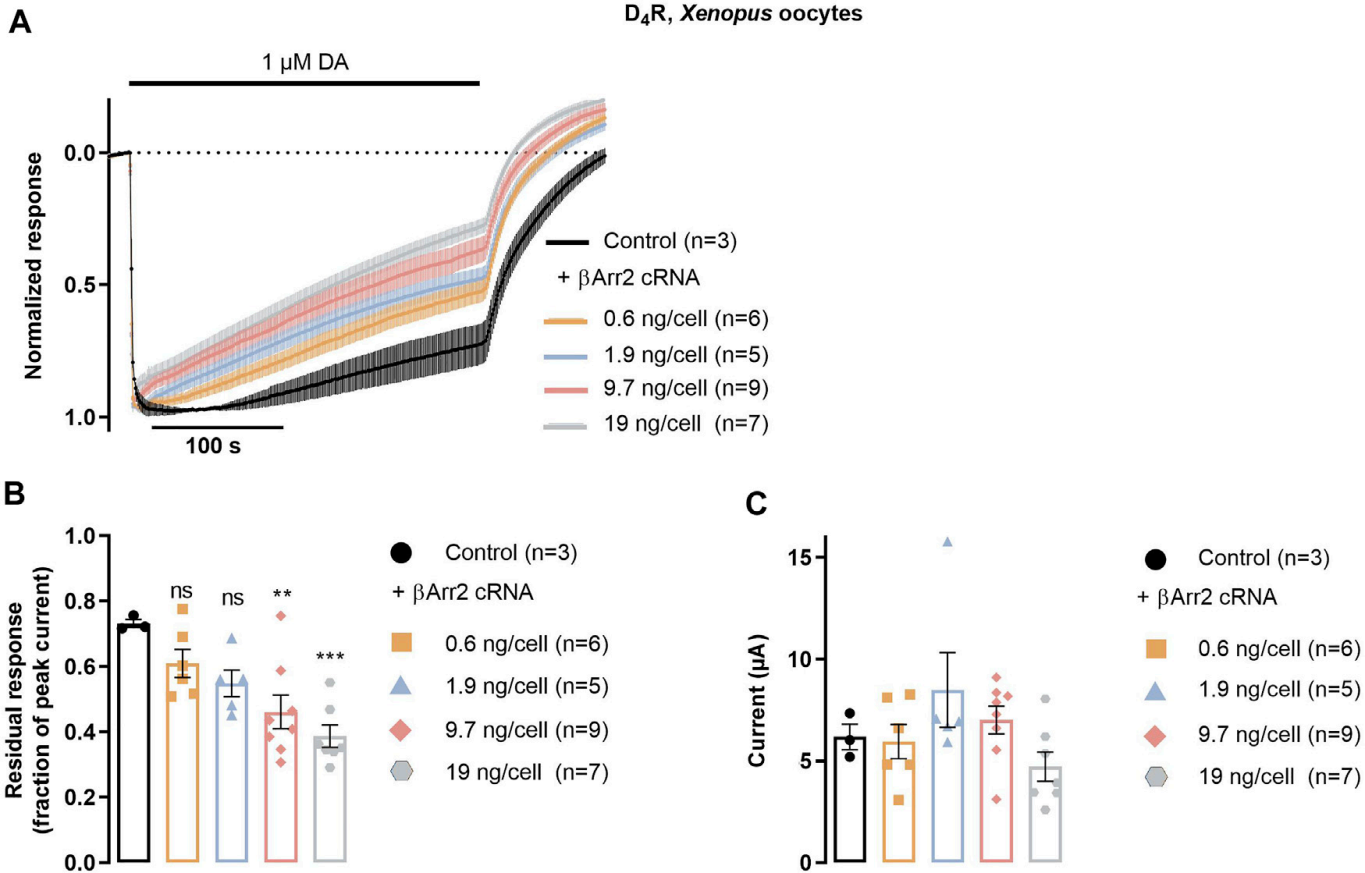
βarr2-induced desensitization of D_4_R in *Xenopus* oocytes. Oocytes were injected with D_4_R cRNA together with RGS4 and GIRK1/4 channel subunit cRNAs, in addition to variable amounts of βarr2 cRNA, as indicated. **(A)** Peak-normalized and averaged traces of GIRK currents evoked by 1 μM dopamine in oocytes co-injected with varying amounts of βarr2 cRNA. **(B)** D_4_R desensitization rate expressed as residual GIRK response (fraction of initial peak response) after 415 s application of 1 µM dopamine. Data from the experiments shown in **(A)** Asterisks denote statistically significant effects of GRK2 coexpression vs control. ***; *p* < 0.001, **; *p* = 0.004, n.s.; not significant, one-way ANOVA with Dunnett’s test for multiple comparisons. **(C)** Peak amplitudes of GIRK current responses shown in **(A)** Data points represent mean ± SEM from three to nine oocytes, as indicated. Superimposed symbols in bar graphs represent individual data points. No statistically significant effects of GRK2 coexpression on current amplitude were observed (Kruskal–Wallis test with Dunn’s correction for multiple comparisons).

To evaluate the functional consequences of agonist-induced βarr2 recruitment to the D_4_R at the level of receptor desensitization, we studied the time course of GIRK currents evoked by the D_4_R in the absence or presence of βarr2. *Xenopus* oocytes are known not to express detectable levels of endogenous arrestins and GRKs (Celver et al., 2013) and therefore provide a suitable background on which to study the effect of exogenous βarr2 and GRK2. Additionally, after injection of the appropriate receptor and channel cRNAs, this system provides a facile readout of D_4_R-mediated GIRK activation (Wedemeyer et al., 2007; Newman-Tancredi et al., 2008; Sahlholm et al., 2008). Upon coexpression of D_4_R, GIRK1/4, and RGS4 with increasing amounts of βarr2 cRNA, a dosage-dependent increase in the rate of decay of dopamine-induced GIRK currents was observed. This effect reached significance for the largest amounts of co-injected βarr2 cRNA (Figures 3A, B), while there was no significant effect on the peak amplitudes of the evoked currents (Figure 3C).

**FIGURE 4:**
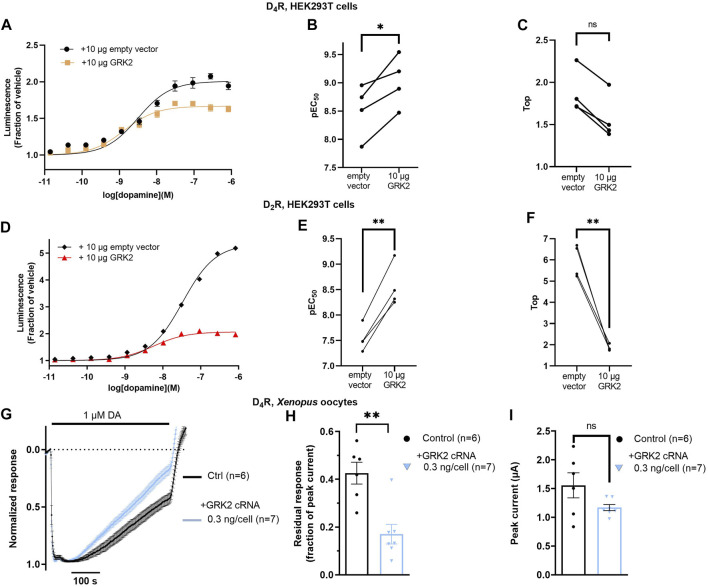
Impact of GRK2 co-expression on arrestin recruitment to the D_4_R and on desensitization of D_4_R-evoked GIRK responses. **(A)** Concentration-dependence of dopamine-induced luminescence increase in cells co-expressing LgBiT- βarr2 and D_4_R-NP, with and without exogenous GRK2 expression. Each data point represents mean ± SEM of data from eight replicate wells. Curves shown from one representative experiment out of a total of four. Data from all experiments are shown in **(B, C)**. **(B)** Co-transfection of 10 µg of GRK2 significantly decreased the dopamine EC_50_ in the βarr2 recruitment assay by about 3-fold. *; *p* = 0.026, Student’s paired *t*-test. **(C)** Co-transfection of 10 µg of GRK2 did not significantly affect the Top parameter. ns; not significant, *p* = 0.125, Wilcoxon matched-pairs signed rank test. **(D)** Concentration-dependence of dopamine-induced luminescence increase in cells co-expressing LgBiT- βarr2 and D_2_R-NP, with and without exogenous GRK2 expression. Each data point represents mean ± SEM of data from eight replicate wells. Curves shown from one representative experiment out of a total of four. Data from all experiments are shown in **(E, F)**. **(E)** Co-transfection of 10 µg of GRK2 significantly decreased the dopamine EC_50_ in the βarr2 recruitment assay by about 10-fold. **; *p* = 0.002, Student’s paired *t*-test. **(F)** Co-transfection of 10 µg of GRK2 significantly decreased the Top parameter. **; *p* = 0.003, Student’s paired *t*-test. **(G)** Peak-normalized and averaged traces of GIRK currents evoked by 1 μM dopamine in oocytes co-injected with D_4_R, RGS4, GIRK1/4, 1.9 ng βarr2, and 0 or 0.3 ng GRK2 cRNAs, as indicated. Data points represent mean ± SEM from six or seven oocytes. **(H)** Residual GIRK responses (fraction of initial peak response) after 415 s of dopamine application, representing the rate of desensitization under the conditions shown in **(G)**. Co-expression of GRK2 significantly decreased the residual response amplitude. **; *p* = 0.005, Mann-Whitney test. **(I)** Peak currents for the conditions shown in **(G)**. Superimposed symbols in bar graphs represent data from individual oocytes. No statistically significant effects of GRK2 coexpression on current amplitude were observed (ns; not significant, *p* = 0.090, Student’s unpaired *t*-test). For some data points in **(A, D)**, SEM bars are obscured by the symbols representing the means.

Finally, we wanted to examine the effect of exogenous GRK2 coexpression on arrestin recruitment to the D_4_R. In the nanoluciferase complementation assay, cotransfection of D_4_R-NP and LgBiT-βarr2 with 10 µg/dish of GRK2-encoding plasmid increased the potency of dopamine by about 3-fold (Figures 4A, B). Observations were paired such that experiments with and without GRK2 were carried out on the same day with cells of the same passage number, transfected 24 h prior. There was a trend toward a decrease in the maximal dopamine-induced luminescence relative to baseline in the GRK2-coexpressing condition; however, this trend did not reach significance (Figure 4C). For comparison, the effect of GRK2 coexpression on βarr2 to the D_2_R was also studied. Cotransfection of D_2_R-NP and LgBiT-βarr2 with 10 µg/dish of the GRK2 plasmid increased the potency of dopamine by ca 10-fold (Figures 4D, E) and significantly reduced the maximal dopamine-induced luminescence increase from about 6-fold to 2-fold (Figure 4F). GRK2 coexpression was not accompanied by any significant change in cell surface FLAG immunoreactivity, neither in cells transfected with D_4_R-NP, nor with D_2_R-NP (Supplementary Figures S1A, B). In electrophysiology experiments in *Xenopus* oocytes, co-injection of GRK2 cRNA with D_4_R, GIRK1/4, RGS4, and βarr2 cRNA accelerated the decay rate of dopamine-induced GIRK currents (Figures 4G, H) as compared to oocytes expressing D_4_R, GIRK1/4, RGS4, and βarr2 in the absence of GRK2, while there was no significant effect on peak amplitudes of the elicited currents (Figure 4I). RGS4 is a GTPase accelerating protein which increases the rate of the G protein cycle and thus causes the state of receptor activity to have a more rapid impact on the GIRK current time course (Doupnik et al., 1997). RGS4 was thus included to increase the measurable rate of desensitization of the GIRK response.

## 4 Discussion

In the present study, we report on the first nanoluciferase complementation assay capable of reporting on dopamine-induced arrestin recruitment to the D_4_R, as well as on the regulation of arrestin recruitment by GRK2. In agreement, the expression of βarr2 induced desensitization of GIRK current responses evoked *via* D_4_R in *Xenopus* oocytes and this desensitization was enhanced by exogenous GRK2. While both β-arrestin1 and β-arrestin2 (βarr2; also known as arrestin3) are expressed in the central nervous system and capable of interacting with D_2_-like receptors, βarr2 has been found to play the major role in D_2_R signaling and desensitization *in vivo* (Beaulieu et al., 2005; Skinbjerg et al., 2009). Here, we therefore chose to study βarr2 together with both D_2_R and the related D_4_R. Likewise, GRK2 has been found to have the strongest effect on D_2_R function of the five non-visual GRKs (Namkung et al., 2009) and we thus focused our present efforts on this GRK isoform. Considering the prominent expression of both βarr2 and GRK2 in the prefrontal cortex (Urs et al., 2016) where D_4_R is also present (Lauzon and Laviolette, 2010), it does not seem unlikely that these proteins may be native D_4_R interaction partners.

We note that some previous investigations that did not detect D_4_R-mediated arrestin recruitment used luciferase complementation (Forster et al., 2020) or fluorescence microscopy to assess redistribution of βarr2 to the plasma membrane (Spooren et al., 2010). On the other hand, some of the studies that did find evidence for such recruitment used PathHunter (Keck et al., 2019; Pirzer et al., 2019) or bioluminescent resonance energy transfer (BRET) approaches to monitor βarr2-D_4_R interactions (Sánchez-Soto et al., 2016; Lyu et al., 2019; Pavletić et al., 2022). Assay sensitivity would seem a likely explanation for these discrepancies. For example, using the brighter nanoluciferase (England et al., 2016) in the present investigation rather than the emerald luciferase employed in a previous complementation study (Forster et al., 2020) may have yielded a stronger signal, allowing an increase in luminescence upon βarr2 recruitment to be picked up. The smaller size of the nanoluciferase fragments and the flexible linker attaching the NP fragment to D_4_R could also mean that there is a greater likelihood of productive interaction (i.e.; enzyme complementation) and less likelihood of steric interference of the enzyme fragments with the interaction between the two tagged proteins.

The *Xenopus* oocyte GIRK assay is another highly sensitive assay that allows for kinetic, live-cell experiments. The dosage-dependent increase in GIRK response decay rate observed with increasing amounts of coinjected βarr2 cRNA suggests that βarr2 mediates desensitization of GIRK responses evoked by D_4_R, similar to what has been described for D_2_R in the corresponding assay (Celver et al., 2013; Ågren and Sahlholm, 2021). In agreement with the results from the nanoluciferase complementation assay, coexpression of GRK2 further increased the rate of response decay.

Consistent with an interaction between D_4_R and GRK2, experiments in HEK cells transfected with D_4_R-NP and GRK2-LgBiT revealed an increase in luminescence upon the addition of dopamine. Compared to cells co-expressing D_4_R-NP and LgBiT-βarr2, this luminescence response peaked and decayed quite rapidly (c.f. Figures 1B, 2B), in the continued presence of dopamine. This behaviour may be interpreted as GRK2 competition for D_4_R binding with endogenous βarr2 (the affinity of which would increase after GRK2 phosphorylation, in line with the results discussed above), a higher affinity of GRK2 for unphosphorylated vs. phosphorylated D_4_R (with GRK2- D_4_R interaction decreasing once the receptor is phosphorylated), or both. Interestingly, the luminescence response in corresponding experiments with D_2_R-NP decayed considerably slower (c.f. Figures 2B, D), suggesting a more sustained interaction between GRK2 and the D_2_R.

The effect of GRK2 coexpression on LgBiT-βarr2 recruitment was more pronounced at the D_2_R compared to the D_4_R, both when considering the increase in dopamine potency and the decrease in maximal arrestin recruitment signal, which reached only a trend level at the D_4_R (Figures 4A–F). This signal reduction was not accompanied by a decrease in FLAG immunoreactivity on intact cells (Supplementary Figure S1), suggesting the cell surface expression of the D_2_R (and of the D_4_R in corresponding experiments) remained similar in the presence or absence of exogenous GRK2. Rather, we would speculate that the decrease in maximal dopamine-induced luminescence signal is due to competition between GRK2 and βarr2 for binding to the receptor, as was also suggested by (Forster et al., 2020), who reported similar findings with regards to GRK2 and βarr2 recruitment to the D_2_R. The stronger effect of GRK2 coexpression on D_2_R signalling would seem consistent with the higher fold-over-baseline increase in luminescence and the slower response decay in experiments with GRK2-LgBiT and D_2_R-NP as compared to D_4_R-NP, as noted above.

## 5 Conclusion

In this study, we presented a nanoluciferase complementation assay able to report on dopamine-mediated βarr2 recruitment to the D_4_R. We also demonstrated that βarr2 recruitment is enhanced by GRK2 coexpression, although this effect is less pronounced than at the D_2_R. In addition, the potentiating action of GRK2 was observed at the level of βarr2-mediated desensitization of GIRK current responses evoked by the D_4_R. Finally, evidence was obtained for transient dopamine-induced recruitment of GRK2 to the D_4_R. The nanoluciferase complementation assay employed here may be useful for drug discovery efforts targeting the D_4_R. In addition, the new information regarding GRK2 regulation of D_4_R may prove relevant for understanding the biological functions of this relatively little-explored dopamine receptor.

## Data Availability

The raw data supporting the conclusion of this article will be made available by the authors, without undue reservation.
