# Supplementary material for: Dopamine-induced arrestin recruitment and desensitization of the dopamine D4 receptor is regulated by G protein-coupled receptor kinase-2
Source: Front Pharmacol. 2023 Jan 27;14:1087171. doi: 10.3389/fphar.2023.1087171 (PMC9911804; doi:10.3389/fphar.2023.1087171)
Supplement: Supplementary file 1 [file Image1.pdf]

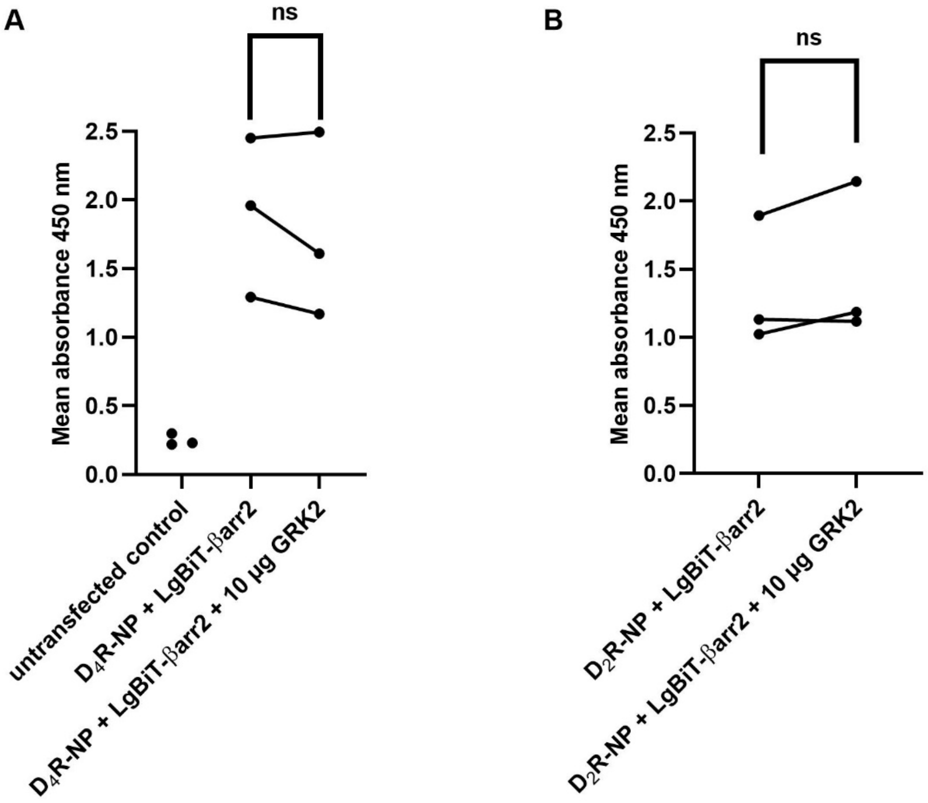

Supplementary Figure 1. Surface expression, as determined by anti-FLAG whole-cell ELISA, of (**A**) D_4R-NP and (**B**) D_2R-NP in HEK 293T cells cotransfected with either receptor construct and LgBit-βarr2 with or without exogenous GRK2. ns; not significant, Student's paired t-test.
